# Preclinical evaluation of [^68^Ga]NOTA-pentixafor for PET imaging of CXCR4 expression in vivo — a comparison to [^68^Ga]pentixafor

**DOI:** 10.1186/s13550-016-0227-2

**Published:** 2016-09-21

**Authors:** Andreas Poschenrieder, Margret Schottelius, Markus Schwaiger, Hans-Jürgen Wester

**Affiliations:** 1 Pharmaceutical Radiochemistry, Technische Universität München, Walther-Meißner-Str.3, 85748 Garching, Germany; 2 Nuklearmedizinische Klinik und Poliklinik, Klinikum rechts der Isar, Technische Universität München, Ismaningerstr. 22, 81675 München, Germany

**Keywords:** GPCR, CXCR4, [^68^Ga]pentixafor, Pentapeptide, NOTA, PET, Radiopharmaceutical, Tracer, Cancer

## Abstract

**Background:**

Due to its overexpression in a variety of tumor types, the chemokine receptor 4 (CXCR4) represents a highly relevant diagnostic and therapeutic target in nuclear oncology. Recently, [^68^Ga]pentixafor has emerged as an excellent imaging agent for positron emission tomography (PET) of CXCR4 expression in vivo. In this study, the corresponding [^68^Ga]-1,4,7-triazacyclononane-triacetic acid (NOTA) analog was preclinically evaluated and compared to the 1,4,7,10-tetraazacyclododecane-1,4,7,10-tetraacetic acid (DOTA) parent compound [^68^Ga]pentixafor.

**Methods:**

NOTA-pentixafor was synthesized by combining solid and solution-phase peptide synthesis. The CXCR4 receptor affinities of [^68^Ga]pentixafor and [^68^Ga]NOTA-pentixafor were determined in competitive binding assays using the leukemic CXCR4-expressing Jurkat T-cell line and [^125^I]FC131 as the radioligand. Internalization and cell efflux assays were performed using CXCR4-transfected Chem-1 cells. Small-animal PET and biodistribution studies were carried out using Daudi-tumor bearing SCID mice.

**Results:**

[^68^Ga]NOTA-pentixafor showed a 1.4-fold improved affinity towards CXCR4 (IC_50_). However, internalization efficiency into CXCR4^+^-Chem-1 cells was substantially decreased compared to [^68^Ga]pentixafor. Accordingly, small-animal PET imaging and biodistribution studies revealed a 9.5-fold decreased uptake of [^68^Ga]NOTA-pentixafor in Daudi lymphoma xenografts (1.7 ± 0.4 % vs 16.2 ± 3.8 % ID/g at 90 min p.i.) and higher levels of non-specific accumulation, primarily in the excretory organs such as the liver, intestines, and kidneys (2.3 ± 0.9 % vs 2.0 ± 0.3 % ID/g, 1.9 ± 0.8 % vs 0.7 ± 0.2 % ID/g, and 2.7 ± 1.1 % vs 1.7 ± 0.9 % ID/g, respectively).

**Conclusions:**

Despite enhanced CXCR4-affinity in vitro, the [^68^Ga]NOTA-analog of pentixafor showed reduced CXCR4 targeting efficiency in vivo. In combination with enhanced background accumulation, this resulted in significantly inferior PET imaging contrast, and thus, [^68^Ga]NOTA-pentixafor offers no advantages over [^68^Ga]pentixafor.

## Background

**Fig. 1:**
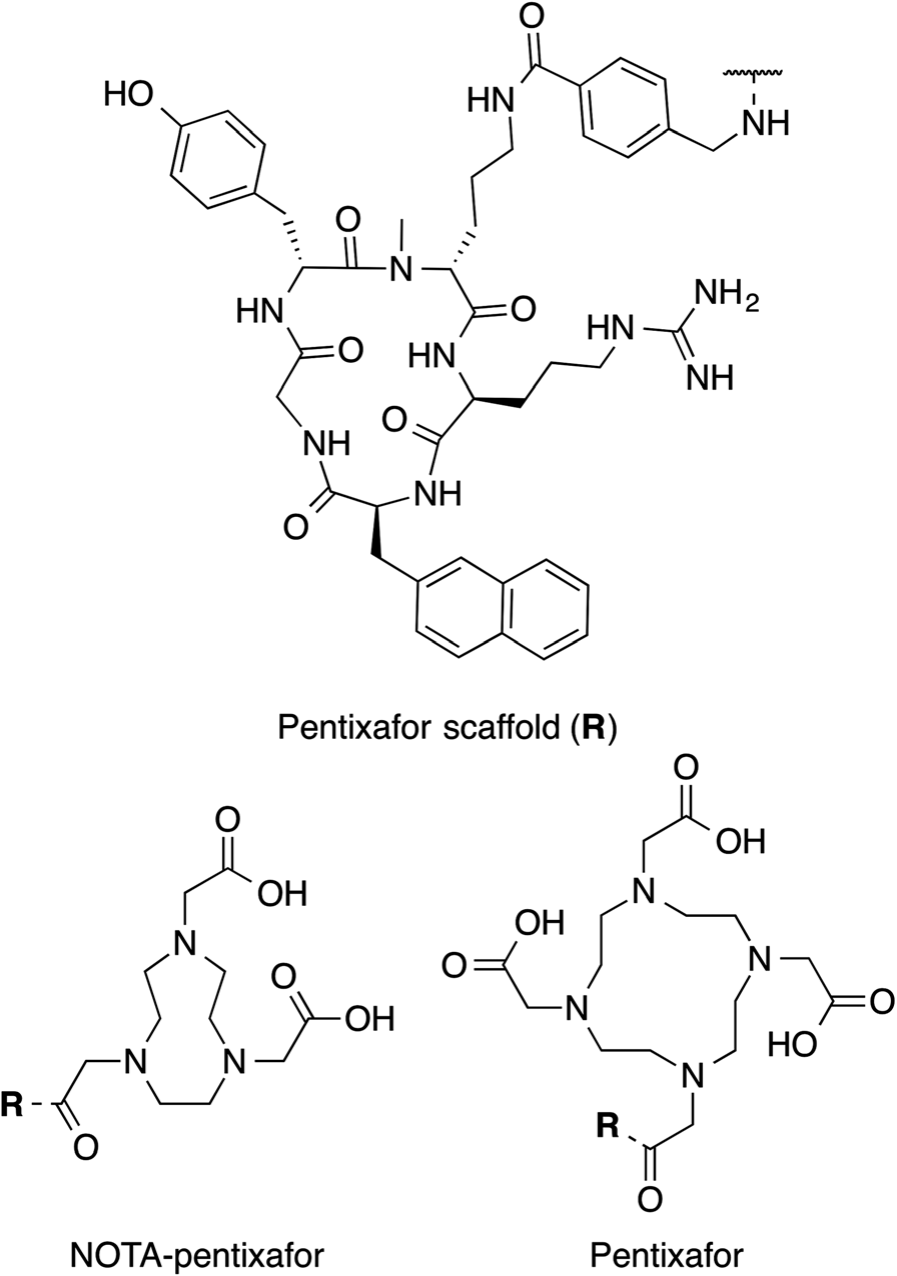
Structures of NOTA-pentixafor (*left*) and pentixafor (*right*)

The chemokine receptor 4 (CXCR4) and its only known natural ligand stromal cell derived factor-1 (SDF-1, CXCL12) have gained considerable attention in oncology, in particular its impact on tumor metastasis [1]. Furthermore, overexpression of CXCR4 has been related to poor prognosis and resistance to chemotherapy [2, 3]. This has led to the development of tools for the non-invasive in vivo quantification of CXCR4 expression in order to improve prognostication and personalized therapy [4]. [^68^Ga]pentixafor, formerly termed [^68^Ga]CPCR4.2 (Fig. 1), represents a milestone in the development of CXCR4-targeted positron emission tomography (PET) probes [5, 6], since its pharmacokinetic properties and favorable dosimetry [7] led to a fast transition into first clinical studies, including in vivo quantification of CXCR4 expression in various types of cancers [8–13] and after myocardial infarction [14–16]. However, triaza-macrocycles like 1,4,7-triazacyclononane-triacetic acid (NOTA) have certain advantages over 1,4,7,10-tetraazacyclododecane-1,4,7,10-tetraacetic acid (DOTA) with respect to chelation of the Ga^3+^ ion [17], e.g. higher thermodynamic stability and kinetic inertness [18–20]. Furthermore, [^nat^Ga]NOTA-pentixafor had already shown improved affinity towards CXCR4 in a previous study [21]. Therefore, [^68^Ga]NOTA-pentixafor was now evaluated preclinically and compared to [^68^Ga]pentixafor (Fig. 1) with respect to its in vivo CXCR4 targeting ability and overall pharmacokinetic profile.

## Methods

General procedures and syntheses of the peptides are described in a recently reported protocol [21]. Determination of tracer lipophilicity [22] and serum stability [23] as well as in vitro studies were performed as recently published [23]. ^68^Ga-labeling of peptides was performed as described using a fully automated system (Scintomics GmbH) [23]; briefly, the ^68^Ge/^68^Ga generator eluate fractions (1.25 mL) were reacted with 5 nmol of peptide. The mixture was buffered with 0.6 mL HEPES (pH = 7.4) to a final pH of 3–4 and heated to 95 °C for 5 min. After purification via one Sep-Pak C8 light cartridge, the ethanolic product fraction was diluted with PBS and used as such for the experiments.

All animal studies were conducted in accordance with the German Animal Welfare Act (Deutsches Tierschutzgesetz, approval No. 55.2-1-54-2532-71-13). For metabolite analysis, 50 MBq of [^68^Ga]NOTA-pentixafor in a total volume of 200 μL of PBS was injected into the tail vein of a CB17 SCID mouse; the animal was sacrificed at 1 h p.i. and blood was collected. After sample preparation, as described in [24], the plasma samples were analyzed by reversed phase (RP)-HPLC and eluate fractions were analyzed using a γ-counter.

For PET (*n* = 3) and biodistribution studies (*n* = 5), an average of 15.2 MBq [^68^Ga]NOTA-pentixafor (100 μL in PBS, 145 pmol, 171 ng) with a specific activity (A_S_) of 104 GBq/μmol was injected intravenously into the tail vein of isofluorane anesthesized female Daudi lymphoma-bearing SCID mice. CXCR4 specificity of tumor accumulation was demonstrated by co-injection of 2 mg/kg AMD3100 (*n* = 3). After static PET imaging for 15 min (Inveon Siemens μPET scanner), the mice were sacrificed (90 min p.i.), and tissues and organs of interest were dissected, weighed, and counted for radioactivity in a γ-counter. The percentage of injected dose per gram of tissue (% ID/g) was calculated; data are shown as mean ± SD.

## Results

[^68^Ga]NOTA-pentixafor was obtained with radiochemical yields of 86.6 ± 3.1 % and a maximal specific activity of 128 GBq/μmol. Radiochemical purities were >99 % as confirmed by radio-TLC. As summarized in Table 1, [^68^Ga]NOTA-pentixafor shows a logP value of −2.4 and is therefore less hydrophilic than its DOTA analog [^68^Ga]pentixafor (logP = −2.9). CXCR4-affinities of the ^nat^Ga-complexed peptides and their metal-free precursors had already been determined previously [21] and are also shown in Table 1. Both peptides show an increased affinity to CXCR4 when metal-labeled, and the ^nat^Ga-NOTA peptide shows slightly improved CXCR4 affinity compared to the ^nat^Ga-DOTA parent compound. Compared to [^68^Ga]pentixafor, total cellular uptake and internalization efficiency of [^68^Ga]NOTA-pentixafor in CXCR4^+^ Chem-1 cells are 2.6- and 7.9-fold decreased, respectively. While 53.3 % of the total cellular activity was found to be internalized for [^68^Ga]pentixafor, only 17.5 % of the total cellular activity was internalized in the case of [^68^Ga]NOTA-pentixafor. Cell efflux studies using [^68^Ga]NOTA-pentixafor revealed intracellular retention of 44.4 ± 0.04 % and 22.7 ± 0.04 % of the initial cellular activity after 0.5 and 1 h, respectively, indicating limited cellular retention of the tracer.

**Table 1:** Comparison of the lipophilicity and the in vitro CXCR4 targeting characteristics of [^68^Ga]NOTA-pentixafor and [^68^Ga]pentixafor

Compound	logP	IC_50_ (nM)	Total cellular activity 1 h (%)	Internalized activity 1 h (%)
NOTA-pentixafor	–	253 ± 49	–	–
[^68^Ga]NOTA-pentixafor	−2.36	17.8 ± 7.7	2.45 ± 0.02	0.43 ± 0.07
Pentixafor	–	102 ± 17	–	–
[^68^Ga]pentixafor	−2.90	24.8 ± 2.5	6.36 ± 0.46	3.39 ± 0.16

Competitive binding studies (IC_50_) were carried out using Jurkat T cells and [^125^I]FC131 as the radioligand. For internalization studies, CXCR4^+^ Chem-1 cells were used

As already observed for [^68^Ga]pentixafor (at 30 min p.i.) [5], metabolite analysis of mouse plasma at 60 min p.i. of [^68^Ga]NOTA-pentixafor revealed the complete absence of radiometabolites and thus demonstrates the metabolic stability of the tracer within the observation period.

Comparative biodistribution data for [^68^Ga]NOTA-pentixafor (*n* = 5) and [^68^Ga]pentixafor (*n* = 6) in Daudi xenograft bearing CB-17 SCID mice (1.5 h p.i.) are summarized in Table 2. Both tracers show rapid clearance from the circulation and predominantly renal excretion. While retention of [^68^Ga]pentixafor in the kidneys is low, [^68^Ga]NOTA-pentixafor shows a 64 % higher kidney uptake. Furthermore, intestinal accumulation of [^68^Ga]NOTA-pentixafor is also 2.8-fold increased compared to the parent compound, most probably due to the increased lipophilicity of the tracer and thus a slightly enhanced hepatobiliary clearance. While the tumor accumulation of [^68^Ga]pentixafor is higher than activity uptake in all other organs, leading to excellent tumor/background ratios (Table 2), uptake of [^68^Ga]NOTA-pentixafor in the Daudi xenografts is surprisingly low, albeit CXCR4 specific. This is illustrated by the competition experiment, where co-injection of 50 μg AMD3100 reduced tumor uptake by 70 %. Due to the low absolute tumor uptake of [^68^Ga]NOTA-pentixafor, however, tumor/organ ratios are <1 for the major excretory organs, suggesting poor imaging contrast.

**Table 2:** Biodistribution data for [^68^Ga]NOTA-pentixafor and [^68^Ga]pentixafor in Daudi xenograft bearing CB-17 SCID mice (1.5 h p.i.) and relating tumor-to-organ ratios

Organ	Tracer		
	[^68^Ga]NOTA-pentixafor (*n* = 5)	[^68^Ga]NOTA-pentixafor + AMD3100 (*n* = 3)	[^68^Ga]pentixafor (*n* = 6)
Blood	0.65 ± 0.41	0.62 ± 0.26	0.97 ± 0.34
Heart	0.34 ± 0.19	0.36 ± 0.14	0.58 ± 0.17
Lung	0.73 ± 0.25	0.95 ± 0.38	1.32 ± 0.29
Liver	2.30 ± 0.88	2.02 ± 0.76	2.05 ± 0.27
Gallbladder	3.61 ± 3.00	5.14 ± 2.24	
Pancreas	0.12 ± 0.05	0.16 ± 0.05	1.06 ± 0.22
Spleen	0.44 ± 0.22	0.54 ± 0.19	0.30 ± 0.10
Kidney	2.71 ± 1.06	3.56 ± 1.03	1.65 ± 0.91
Adrenals	0.52 ± 0.30	0.48 ± 0.15	3.68 ± 0.72
Stomach	0.45 ± 0.21	0.58 ± 0.24	0.48 ± 0.08
Intestine	1.89 ± 0.83	3.97 ± 0.92	0.67 ± 0.21
Muscle	0.13 ± 0.10	0.13 ± 0.05	0.19 ± 0.06
Bone	0.25 ± 0.18	0.16 ± 0.07	n/a
Tumor	1.71 ± 0.40	0.52 ± 0.17	16.2 ± 3.82
Tumor-to-organ ratio			
T/blood	2.64 ± 1.78		16.7 ± 7.05
T/liver	0.74 ± 0.34		7.90 ± 2.13
T/kidney	0.63 ± 0.29		9.81 ± 5.89
T/muscle	13.0 ± 9.86		85.2 ± 33.6

CXCR4 specific tumor accumulation of [^68^Ga]NOTA-pentixafor was demonstrated by co-injection of 50 μg AMD3100. Data are given in % ID/g tissue and are means ± SD

**Fig. 2:**
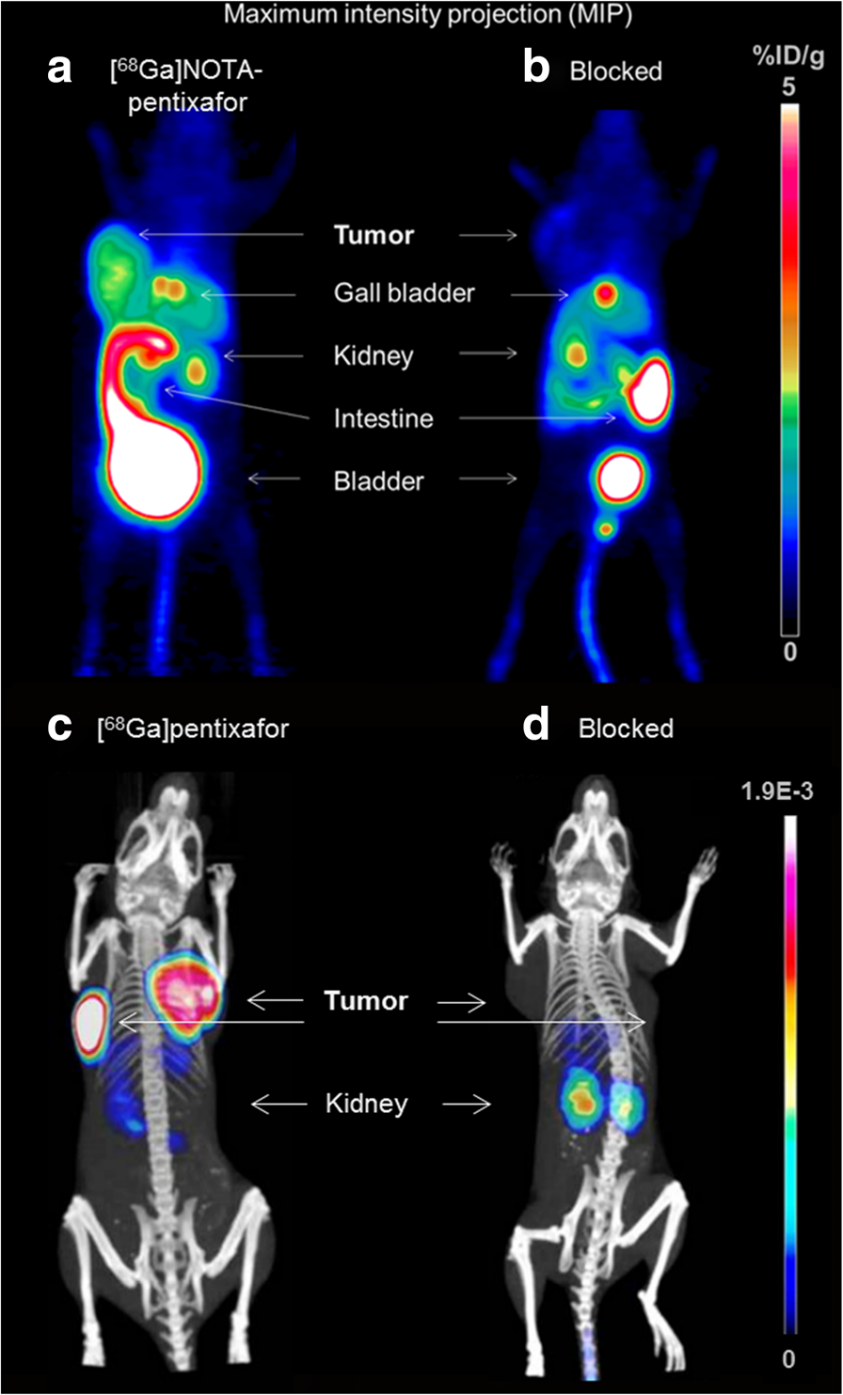
**a**, **b** μPET images of Daudi xenograft bearing CB-17 SCID mice at 90 min p.i. of [^68^Ga]NOTA-pentixafor (15.2 MBq, 195 pmol/171 ng peptide; **a** tracer only; **b** co-injection with 50 μg AMD3100). **c**, **d** μPET/CT images of Daudi (*left*, high CXCR4) and SU-DHL-8 (*right*, low CXCR4) lymphoma xenografts at 90 min p.i. of 5 MBq [^68^Ga]pentixafor (**c**) and co-injection of 50 μg AMD3100 (**d**). Bladder activity was blanked out [12]. Credit **c** and **d**: © 2015 Ivyspring International Publisher

This was confirmed by small-animal PET imaging studies. Representative PET images of both tracers are shown in Fig. 2. As expected from the biodistribution data, [^68^Ga]NOTA-pentixafor uptake in the Daudi xenograft was CXCR4 specific (Fig. 2b), and despite low total activity accumulation, tumors were clearly delineated 1.5 h p.i. In contrast to [^68^Ga]pentixafor, however, which shows virtually no background accumulation except in kidneys [12], [^68^Ga]NOTA-pentixafor also shows considerable activity accumulation in the gall bladder, intestines, and kidneys.

## Discussion

We recently reported the influence of different metal-chelate conjugates of pentixafor on the CXCR4 affinity [21] and found that the NOTA conjugate NOTA-pentixafor (Fig. 1) displayed the highest CXCR4 affinity among various tracers. Because NOTA offers a better suited coordination cavity for Ga^3+^ incorporation and higher thermodynamic stability and kinetic inertness compared to DOTA [18–20], [^68^Ga]NOTA-pentixafor was now evaluated preclinically and compared to [^68^Ga]pentixafor (Fig. 1), which is currently entering clinical studies for PET-based quantification of CXCR4 expression in vivo [5, 6, 12]. Surprisingly, despite improving CXCR4 affinity of the ligand, the exchange of DOTA by NOTA had deleterious effects on overall pharmacokinetics, both with respect to CXCR4 targeting efficiency and clearance characteristics. This is mainly attributed to the differences in chelator denticity, overall charge, and resulting changes in the complex geometry, which also affects the lipophilicity (Table 1). The increased logP of [^68^Ga]NOTA-pentixafor seems to be the main reason for the enhanced background accumulation of the new compound, especially in the gallbladder and intestines. Moreover, the [Ga]NOTA-for-[Ga]DOTA exchange within the pentixafor conjugates alters the overall charge of the chelator moiety from neutral to positive which can also influence the pharmacokinetic profile. The strong dependence of the pharmacokinetics on the chelator and radiometal have also been reported for somatostatin [25, 26] or bombesin-targeting peptides [27]. In contrast, the unexpectedly low tumor accumulation of [^68^Ga]NOTA-pentixafor in the Daudi xenograft model may be mainly attributed to the substantially decreased internalization efficiency of [^68^Ga]NOTA-pentixafor compared to [^68^Ga]pentixafor, which also seems affected by the structural changes induced by the NOTA-for-DOTA substitution. Such substantial influence of the chelator on the biodistribution has also been shown in human epidermal growth factor receptor type 2 (HER2)-targeting affibodies with DOTA, NOTA, or NODAGA-conjugates [28] as well as other GPCR ligands such as somatostatin receptor-targeting ^68^Ga-labeled [Tyr^3^]octreotide [25].

## Conclusion

Despite improved CXCR4 affinity, [^68^Ga]NOTA-pentixafor showed severely compromised CXCR4 targeting efficiency compared to the parent compound [^68^Ga]pentixafor, both in vitro and in vivo. Alongside, a substantially decreased uptake in CXCR4-positive lymphoma xenografts, [^68^Ga]NOTA-pentixafor also shows enhanced accumulation in the excretory organs, leading to low tumor/background ratios and inferior imaging contrast compared to [^68^Ga]pentixafor. The present data on [^68^Ga]NOTA-pentixafor underline the strong dependence of the pharmacokinetics of pentixafor-based peptides on the chelator and radiometal and highlight the outstanding characteristics of [^68^Ga]pentixafor for successful CXCR4 imaging.

## Abbreviations

A_S_: Specific activity
CXCR4: Chemokine receptor 4
DOTA: 1,4,7,10-tetraazacyclododecane-1,4,7,10-tetraacetic acid
HER2: Human epidermal growth factor receptor type 2
IC_50_: Half maximal inhibitory concentration
NOTA: 1,4,7-triazacyclononane-triacetic acid
p.i.: Post injection
PET: Positron emission tomography
SDF-1: Stromal cell derived factor-1
